# *T* and *Z*, partial seed coat patterning genes in common bean, provide insight into the structure and protein interactions of a plant MBW complex

**DOI:** 10.1093/g3journal/jkae184

**Published:** 2024-08-21

**Authors:** Phillip E McClean, Jayanta Roy, Christopher L Colbert, Caroline Osborne, Rian Lee, Phillip N Miklas, Juan M Osorno

**Affiliations:** Department of Plant Sciences, North Dakota State University, Fargo, ND, USA 58108; Genomics, Phenomics, and Bioinformatics Program, North Dakota State University, Fargo, ND, USA 58108; Department of Plant Sciences, North Dakota State University, Fargo, ND, USA 58108; Department of Chemistry and Biochemistry, North Dakota State University, Fargo, ND, USA 58108; Department of Plant Sciences, North Dakota State University, Fargo, ND, USA 58108; Genomics, Phenomics, and Bioinformatics Program, North Dakota State University, Fargo, ND, USA 58108; Department of Plant Sciences, North Dakota State University, Fargo, ND, USA 58108; Legume Genetics and Physiology Research Unit, USDA-ARS, 24106 N. Bunn Rd., Prosser, Washington, USA 99350; Department of Plant Sciences, North Dakota State University, Fargo, ND, USA 58108

**Keywords:** AlphaFold2, bHLH, common bean, MBW, MYB, protein interactions, protein modeling, WDR

## Abstract

Flavonoids are secondary metabolites associated with plant seed coat and flower color. These compounds provide health benefits to humans as anti-inflammatory and antioxidant compounds. The expression of the late biosynthetic genes in the flavonoid pathway is controlled by a ternary MBW protein complex consisting of interfacing **M**YB, **b**eta-helix–loop–helix (bHLH), and **WD**40 **R**epeat (WDR) proteins. *P*, the master regulator gene of the flavonoid expression in common bean (*Phaseolus vulgaris* L.), was recently determined to encode a bHLH protein. The *T* and *Z* genes control the distribution of color in bean seeds and flowers and have historically been considered regulators of the flavonoid gene expression. *T* and *Z* candidates were identified using reverse genetics based on genetic mapping, phylogenetic analysis, and mutant analysis. Domain and AlphaFold2 structure analyses determined that *T* encodes a seven-bladed β-propeller WDR protein, while *Z* encodes a R2R3 MYB protein. Deletions and SNPs in *T* and *Z* mutants, respectively, altered the 3D structure of these proteins. Modeling of the Z MYB/P bHLH/T WDR MBW complex identified interfacing sequence domains and motifs in all three genes that are conserved in dicots. One Z MYB motif is a possible beta-molecular recognition feature (β-MoRF) that only appears in a structured state when Z MYB is modeled as a component of a MBW complex. Complexes containing mutant T and Z proteins changed the interaction of members of the complex in ways that would alter their role in regulating the expression of genes in the flavonoid pathway.

## Introduction

Common bean (*Phaseolus vulgaris* L.) seeds are rich in many nutrients important for human health and well-being. The multiple dry bean market classes differ in their concentration of nutrients, such as flavonoids that impart the various colors found unique to each market class (Madrera and Valles 2020). The master regulator of all color expressions is the *P* (*=Pigment*; Emerson 1909) gene. Any homozygous recessive *pp* genotype has white seed coats and flowers. The seed coat color is controlled by multiple genes, including *G*, *B*, *V*, *Rk*, *R*, and *Sal* (see Bassett 2007 for review). These genes were assumed to encode either enzymes or regulators of the flavonoid pathway. This was found to be the case for *V*, which was determined to encode flavonoid 3′5 ′ hydroxylase (McClean *et al*. 2022).

**Fig. 1.**
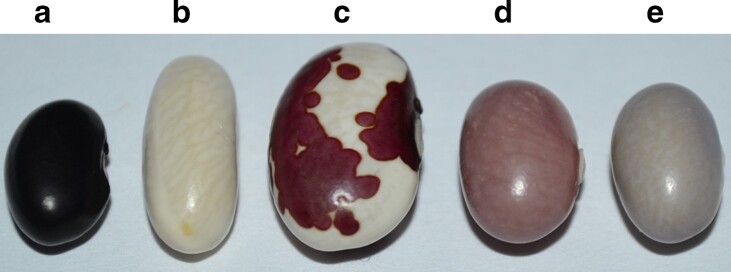
Germplasm used to map the *T* and *Z* genes of common bean. The *T* and *Z* alleles for each genotype are in parenthesis. a) 5-593 (*T Z*). b) Earliwax (*t*^EW^*z*^EW^). c) 65–73 (*t*^65–73^*Z*). d) V0869 (*T z*^EW^). e) V0919 (*T z*^EW^).

Common bean is somewhat unique since its seed coat can be either solid colored, dark striped, or mottled often over a light tan background or partially colored with white and colored zones. The stripe/mottle patterns are controlled by the recessive alleles of genes in the *C* locus (Prakken 1974). The partial seed coat types are distinct from the patterned types, with color restricted to a specific zone of the seed coat, while the remainder of the seed coat is white. All partial seed coat patterns are controlled by a second genetic system, which requires a homozygous recessive *tt* genotype at the *T* (*=Total*; Emerson 1909) gene. Seeds with dominant *P*, *T*, and *C* alleles are solid colored (Fig. 1a). The extent of the colored region is controlled by the interaction of the *tt* genotype and various alleles at the *Z* (=*Zonal*; Tschermak 1912), *Bip* (=*Bipunctata*; Lamprecht 1932), *J* (=*Joker*; Lamprecht 1932), and *Fib* (=*fibula*; Bassett 2001) genes. For example, *P t Z Bip J fib* genotypes express full seed color, while *P t z j*^ers^ seeds are white (Fig. 1b), and *P t z Bip J fib* express dorsal seed color (virgarcus pattern, Fig. 1c). *Z* and *J* also control the expression of color in the hilum ring, a layer of cells bordering the hilum. In a *P T z j* background, color is not expressed in the hilum ring (see a detailed summary in Bassett 2007). The effect of *T* and *Z* and other partial seed coat genes on the spatial distribution of color led to the suggestion that many if not all of these genes are regulators of the flavonoid pathway.

*P*, the first gene to be described as a molecular component of color gene regulation in bean, encodes an ortholog of other beta-helix–loop–helix proteins (bHLH; McClean *et al*. 2018) involved in flavonoid bioisynthesis. It functions in a ternary complex along with a WD40 repeat (WDR) protein and one of several MYB proteins. This complex, termed MBW, regulates the late biosynthetic genes (LBGs) of the flavonoid pathway (Stracke *et al*. 2007; Stracke *et al*. 2010). The WDR protein is encoded by a single gene in most species; one or a few genes encode the bHLH protein; and one of multiple MYB activators or repressors interacts with its two complex partners to fine-tune expression of the flavonoid LBGs. The MBW complex involves several protein interactions. The MYB and bHLH proteins were initially shown to physically interact (Goff *et al*. 1992) and bind upstream of the flavonoid LBGs (Goff *et al*. 1990). The bHLH and WDR proteins initially interact and form a ternary complex with a MYB factor (Payne *et al*. 2000; Zhang *et al*. 2003). WDR and MYB factors were reported to interact in some (Liu *et al*. 2018; Cui *et al*. 2021), but not all species (Gao *et al*. 2018). Mutations of any member of the MBW complex directly affect the expression of the flavonoid pathway LBGs and alter seed coat flavonoid composition. For example, Arabidopsis mutants of the MBW MYB gene (*TT2*; Nesi *et al*. 2001) have golden yellow testa, while mutants of the bHLH (*TT8*; Nesi *et al*. 2000) and WDR (*TTG1*; Walker *et al*. 1999) genes have colorless testa. In a similar vein, mutants of the common bean *P* gene, the common bean ortholog of the Arabidopsis bHLH *TT8* gene, have colorless seed coats (McClean *et al*. 2018). MBW genes from other species also have a strong effect on seed coat color expression. Three legume genes, pea *A2* (Hellens *et al*. 2010), *Medicago truncatula* MtWD40-1 (Pang *et al*. 2009; Meng *et al*. 2019), and *Vicia faba zt1* (Gutierrez and Torres 2019), encode WDR proteins, and mutants of these genes express white flowers and seeds.

Protein complexes function through defined physical interactions of partners. An understanding of the interactions among the MBW partners is necessary to understand how the complex regulates flavonoid expression. Regulation of the pathway often involves altering the physical interaction of the MYB partner with its bHLH and WDR partner (Chen *et al*. 2019). As demonstrated in *Brassica rapa*, the MBW complex can be disrupted by a single amino acid change in the MYB partner, which in turn inactivates the complex by eliminating its interaction with the bHLH protein (Yang *et al*. 2021). In peach, a repressor protein, PpMYB18, passively competes with the MYB activator to bind to the bHLH component of the complex (Zhou *et al* 2019). Arabidopsis MYBL2, a R3 MYB protein, was identified as a repressor of proanthocyanidin synthesis (Dubos *et al*. 2008) and later shown to disrupt the MBW complex by binding to the bHLH partner (Thevenin *et al*. 2012). Most recently, AP2, a regulator of seed development, was found to bind to MYBL2, and the AP2–MYBL2 complex interacts with the bHLH protein and effectively disrupts the MBW complex and inhibits the expression of the LBGs (Jiang *et al*. 2023). Flavonoid biosynthesis requires a carbon source, and under low sucrose conditions, the MBW complex formation is repressed. From a developmental perspective, sucrose levels are monitored by the SnRK1 kinase regulatory complex. Recent evidence found that under carbon stressed conditions, SnRK1 represses the expression of the MYB component and phosphorylates MBW partners, which leads to complex dissociation (Broucke *et al*. 2023).

The partial seed coat pattern phenotype provides a defined genetic system not fully elucidated in other species to understand the spatial regulation of the flavonoid pathway. The goal here is to identify candidate genes for the common bean *T* and *Z* genes. *T* is a focus because it is the essential regulator for any partial seed coat pattern. *Z* is a focus because *tt* mutants further restrict color expression beyond that of *tt* genotypes. Since these mutants result in zones without color expression, and that a functioning MBW complex is required for color expression, the further assumption is that *T* and *Z* function act as regulators of flavonoids and are possibly components of the MBW complex. Here, genetic analysis, candidate gene identification, phylogenetic comparisons, and protein modeling identified a β-propeller, WDR-encoding gene as a strong candidate gene for the *T* gene. Using the same experimental approaches, a MYB-encoding gene model was identified as a strong *Z* candidate gene. As a first step in understanding how these two proteins might physically interact as part of bean MBW complex, wild-type and mutant versions of the *T*, *Z*, and *P* genes were modeled by the recently released AlphaFold2 (AF2) protein modeling algorithms (Jumper *et al*. 2021). Additionally, using dimers and trimers of the T, Z, and P proteins, a MBW complex was modeled for the first time to define specific interacting domains among the three wild type proteins and the observed effects of mutant proteins on those interactions. These results provide an initial framework to further investigate the complex further with other experimental procedures.

## Material and methods

### Plant material

Seed stocks used for the genetic component of the research were obtained from USDA National Plant Germplasm System (https://www.ars-grin.gov/Collections#plant-germplasm). These are designated with a PI number. 5-593 (PI 608674) is a genotype selected by Dr. Mark Bassett (University of Florida) from a genetic stock developed by Dr. Jim Beaver (University of Puerto Rico). 5-593 is dominant for nearly all seed coat color and pattern genes. The 5-593 (Fig. 1a) genotype for the patterning and color genes is *T Z Bip P* [*C r*] *J G B V Rk Gy sal.* Dr. Bassett introgressed recessive alleles for many color and pattern genes into 5-593 through a backcrossing program to the level of BC_3_. Candidate recessive *t* and/or *z* alleles were sequenced from the following donor lines: 65–73 (PI 451802; Fig. 1c), homozygous recessive for a t allele; V0869 (PI 527806; Fig. 1d) and V0919 (PI 527820; Fig. 1e), homozygous for recessive *z* and Earliwax (PI 549618; Fig. 1b), and homozygous recessive for both *t* and *z* (Fig. 1). Dr. Bassett's genetic marker introgression lines and donor genotypes for the mutant alleles in the introgression lines were evaluated for sequence variability with *T* and *Z* PCR Allele Competitive Extension (PACE) markers for the two genes (Supplementary Table 1). The two PACE markers were also used to determine the allelic state of these two genes in a wide collection of genotypes from the Middle America Diversity Panel [MDP (Moghaddam *et al*. 2016; Supplementary Table 2] and Andean Diversity Panel [ADP (Cichy *et al*. 2015; Supplementary Table 3].

### DNA isolation, amplicon sequencing, and CDS assembly

DNA was extracted from young leaf tissue using the Mag-Bind Plant DNA Plus Kit (Omega Bio-Tek; https://www.omegabiotek.com/product/mag-bind-plant-dna-plus-96-kit/). DNA amplicons were generated by PCR amplification with the primers listed in Supplementary Table 4 and the PCR protocol described in McClean *et al*. (2022). The reaction was performed in a 25-µl volume over 45 cycles using the annealing temperatures specific for each pair of primers. The NEB Monarch Gel Extraction Kit (https://www.neb.com/products/t1020-monarch-dna-gel-extraction-kit) was used to isolate individual amplicon fragments, and individual fragments were sequenced by Eton Bioscience Inc. (https://www.etonbio.com/). The coding region of the fragment sequences were assembled using the Staden Package (https://sourceforge.net/projects/staden/; Staden 1996) into the CDS sequence. The NCBI T proteins identifiers (genotype in parentheses) are OR500718(PI 451802), OR500719 (PI 549618), OR500720 (PI 632734), and OR500721 (PI 608674). The NCBI Z protein identifiers (genotype in parentheses) are OR475097 (PI 549618) and OR475098 (PI 608674).

### Phylogenetic tree building and T and Z gene alignment of orthologs

A neighbor-joining (NJ) phylogenetic tree was constructed with the T protein (Pv5-593.09G047300) and T ortholog proteins that represent the breadth of angiosperm diversity. The orthologs were identified via blastp search of Phytozome 13 (https://phytozome-next.jgi.doe.gov/). The *Z* NJ tree consisted of Pv5-593.03G127600 and functionally verified flavonoid activator and repressor MYB proteins identified from the literature. Each NJ tree was built using MEGA 7 (Kumar *et al*. 2016). Tree building was based on the MUSCLE protein alignment algorithm (Edgar 2004). The Jones–Taylor–Thornton substitution model was used for tree development. The NJ tree was obtained using 1,000 bootstrap replicates. Protein alignment was performed using the T-Coffee server (http://tcoffee.crg.cat/apps/tcoffee/do:regular), and the alignments were colored using MS Word.

### PCR Allele Competitive Extension (PACE) marker development

PACE markers were developed for the candidate *T* gene mutations discovered in Earliwax (1-bp deletion) and 65–73 (39-bp deletion). The nucleotide deletion in Earliwax is at position Pv09:10,713,827. The 39-bp deletion 65–73 begins at position PV09:10,713,103 bp. PACE markers were also developed for two Earliwax mutations in the *Z* gene candidate. One mutation was an SNP at position Pv03:34,523,093 bp, and the second mutant was a deletion in the interval Pv03:34,522,869..Pv03:34,522,880 bp. Each position is relative to the 5-593 reference genome assembly (https://phytozome-next.jgi.doe.gov/info/Pvulgaris5_593_v1_1). For each polymorphism, the unique forward and common reverse primers (Supplementary Table 5) were designed by the 3CR Bioscience (https://3crbio.com/) primer development service. The genotyping reactions contained 2 µl (of approximately 10 ng/ul) of DNA, 0.15 µl of primer mix (12 µM of each allele-specific forward primer, 30 µM of the common reverse primer), 4 µl of 3CR Bioscience PACE Master Mix Standard Rox, and 1.85 µl H_2_O for a total volume of 8 µl. PCR amplifications were performed with the following PCR cycling conditions: 94°C for 15 min, followed by a touchdown profile of 10 cycles at 94°C for 20 s, and 65°C for 1 min with a 0.8°C reduction per cycle, followed by 40 cycles at 94°C for 20 s and 57°C for 1 min. End point reads for the 96 well plates were collected using the Bio-Rad CFX96 Touch Real-time PCR Detection System to determine the allele of each genotype. Readings were made after PCR, and the relative fluorescent unit (RFU) data were output using the Bio-Rad CFX Maestro 2.0 software.

### Genetic mapping of the T WDR and Z MYB candidate genes and variation in common bean germplasm

F_2_ populations (Brady *et al*. 1998; Bassett *et al*. 1999) based on crosses of genetic stocks developed by Dr. Bassett (Univ. of Florida) were used to map candidate genes relative to the *T* and *Z* phenotypes. PACE markers were developed that span the interval defined by the physical position of OM19_400_ and AM10_560_, two RAPD markers determined by Brady *et al*. (1998) to be linked to *T* and *Z*, respectively. One F_2_ population (4–68) was used to map *T* with the new markers and its relationship to the original *T* marker. The *Z* gene was mapped relative to the *Z* marker position in three F_2_ populations (4–76, 6–273, and 6–160.161). The cross, donor recessive allele, and phenotypic ratios can be found in Supplementary Table 6.

### Protein sequence analysis and structure predictions of the bHLH P protein, the WDR T gene protein, and the MYB Z gene protein

The domain structure of the Pv5-593.09G047300 and Pv5-593.03G127600 proteins was determined with the SMART application (http://smart.embl-heidelberg.de/), as implemented in Interpro Paysan-Lafosse *et al.* 2022; (https://www.ebi.ac.uk/interpro/). Intrinsically disordered regions (IDR) of the P bHLH, Z MYB, and T WDR proteins were determined using the IUPred3 (Erdős *et al*. 2021) and DISOPRED3 (Jones and Cozzetto 2015) algorithms. Regions with disorder scores greater than 0.5 were considered to be disordered.

For protein structure predictions, the AlphaFold2 (AF2; Jumper *et al*. 2021) neural network, as implemented in ColabFold (Mirdita *et al*. 2022; https://github.com/sokrypton/ColabFold), was used for all protein structural predictions. Protein complex predictions were generated using Alphafold-Multimer (AF-Multimer; Evans *et al*. 2021), also as implemented in ColabFold. MMseqs2 (Steinegger and Söding 2017) was used for the homolog search against the UniRef90 database. Five models were developed, and the model with the highest predicted local distance difference test (pLDDT) score was selected as the best model. The predicted protein structure was visualized using ChimeraX (Pettersen *et al*. 2021). The structural interaction of the modeled bean MBW complex was analyzed with the “Protein interfaces, surfaces and assemblies’ (PISA) service at the European Bioinformatics Institute (https://www.ebi.ac.uk/pdbe/pisa/; Krissinel and Henric 2007). The ChimeraX Matchmaker function was used to calculate the least-squares-fit root–mean–square deviation (RMSD) between any two predicted structure models.

## Results and discussion

### Identifying *T* and *Z* candidate genes

**Fig. 2.**
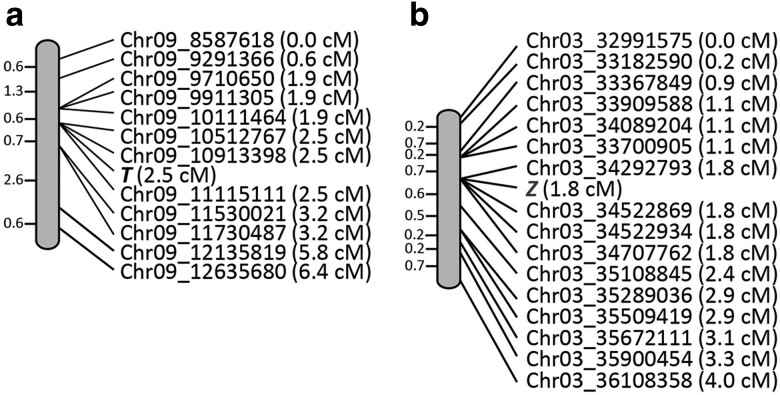
Genetic maps of *T* and *Z* developed using PACE SNP markers bordering the position of RAPD markers linked to each gene. a) The *T* gene was mapped in population 4–68 (Brady et al. 1998). b) The *Z* gene was mapped in three populations (4–76, 6–273, and 6–160.161). For both a) and b), the physical position of the polymorphic SNP follows the chromosome designation, and the genetic position is located in parenthesis.

*T* cosegregates with marker OM19_400_, while *Z* is closely linked with marker AM10_560_ (Brady *et al*. 1998). These genes were then mapped to chromosomes Pv09 and Pv03, respectively (McClean *et al*. 2002). Blastn analysis identified the location of the markers in the 5-593 genome. Based on the ratio of genetic to physical distance in this region of the genome, the *T* gene was located to a 10.11–11.53 Mb interval on chromosome Pv09 (Fig. 2a), while *Z* was mapped to the 33.70–35.11 Mb interval on Pv03 (Fig. 2b).

Based on the strong phenotypic effects of *T* and *Z* on color expression in seed coats and flowers, a search within each interval was performed to identify gene models predicted to encode a protein with a flavonoid pathway enzymatic (Wu *et al*. 2018) or regulatory (Zhang and Schrader 2017) function. A *T* gene candidate, *Pv5-593.09G047300*, was identified in the interval and encodes a WDR protein that begins at position 10,712,956 bp of the 5-593 reference genome sequence (Fig. 2a). Based on replicated RNA-seq data for 5-593 available at Phytozome, the gene was expressed in four progressive stages of seed coat development. A blastp analysis found that Pv5-593.09G047300 was orthologous to the Arabidopsis *TTG1* gene (AT5G24520) that also encodes a WDR protein (Walker *et al*. 1999) and anchors the TT8 βHLH in the MBW complex. Pv5-593.09G047300 is also orthologous to the *Medicago truncatula* MtWD40-1 WDR protein (Medtr3g092840) that controls seed coat color expression (Pang *et al*. 2009), the *Pisum sativum* A2 WDR protein (NCBI identifier ADQ27318.1) that controls flower color (Hellens *et al*. 2010), and *Glycine max* GmWD40 (Glyma.06G136900) that is required to activate proanthocyanin production (Lu *et al*. 2021). MtWD40-1 was recently confirmed as the WD40 component of the *M. truncatuala* MBW complex (Meng *et al*. 2019). A multi-sequence alignment found the sequences of these five proteins to be highly conserved (Supplementary Fig. 1).

Gene model *Pv5-593.03G127600*, which encodes an R2R3-MYB protein located within the *Z* gene interval, was identified as a *Z* candidate gene using the same approach as with *T*. Replicated RNA-seq analysis found the gene to be expressed only during the four stages of seed coat development. Pv5-593.03G127600 was orthologous to the Arabidopsis TT2 protein (Nesi *et al*. 2001), one of the several MYB factors that functions in the Arabidopsis MBW complex. Pv5-593.03G127600 was also orthologous to the *M. truncatula* MtMYB14 protein that interacts with another R2R3-MYB protein, MtMYB5, to activate LBGs of the flavonoid pathway (Liu *et al*. 2014). This activation is in conjunction with MBW proteins MtTT8 (a bHLH ortholog of the Arabidopsis TT8 protein and the common bean *P* gene) and MtWD40-1 (a WDR ortholog of Arabidopsis TTG1 protein). The blastp analysis also identified two orthologs in the Williams82 reference soybean genome, Glyma.13G109100 and Glyma.17G050500, that were labeled GmTT2A and GmTT2B, respectively (Lu *et al*. 2021). These proteins interact with the soybean bHLH and WDR proteins to activate expression of flavonoid LBGs (Lu *et al*. 2021). Overexpression of *Glyma.17G050500* in transgenic soybeans resulted in greater flavonoid pigmentation in the hilum. Similarly, *Z* also controls hilum pigmentation in common beans (Bassett *et al*. 1999). The sequence alignment of the candidate Z protein, along with the AtTT2, MtMYB14, and GmTT2A proteins, found a high degree of conservation within the N-terminal R2 and R3 MYB domains, while the C terminal region was much less conserved (Supplementary Fig. 2).

### Phylogenetic analyses of T WDR and Z MYB and their orthologs

**Fig. 3.**
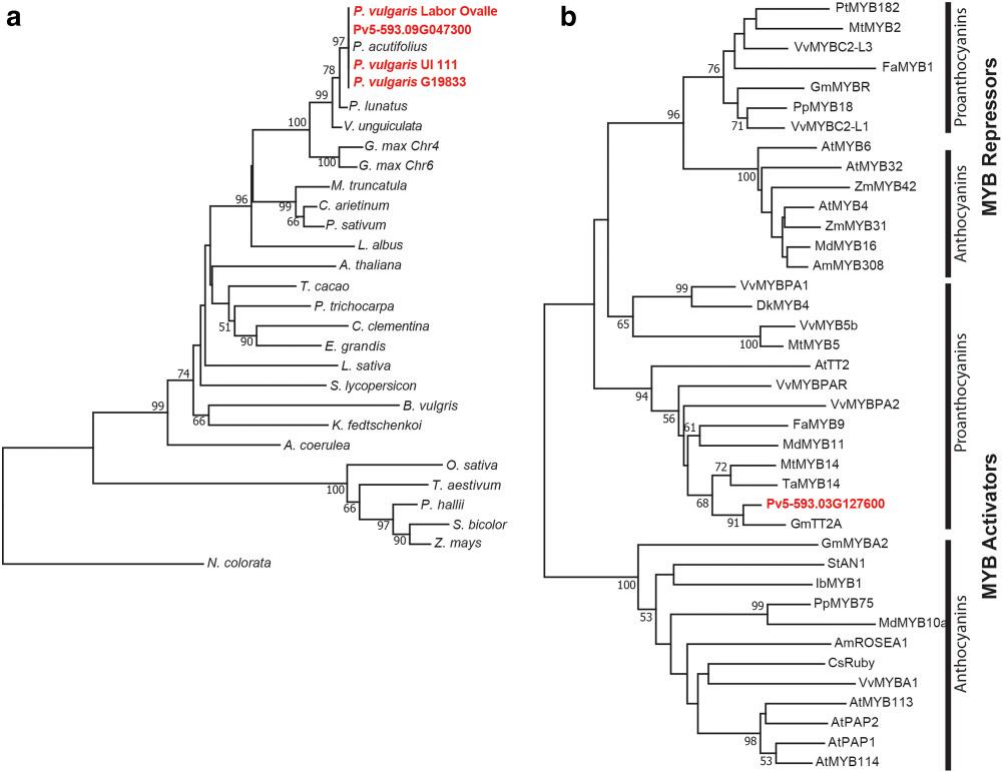
Neighboring joining trees of WDR homologs of common bean a) angiosperm WDR proteins and b) MYB proteins experimentally to act as repressors or activators of flavonoid biosynthesis. Common bean gene models are shown in red.

A blastp analysis across the *Phaseolus* genus and 22 other species representing the taxonomic breadth of the Angiosperms was performed. The T WDR (Pv5-593.09G047300) protein sequence was identical to its orthologs from the other three bean reference genome annotations (G19833 v2.1, UI111 v1.1, and Labor Ovalle v.1.1), tepary bean (*P. acutifolius*), and scarlet runner bean (*P. coccineus*). T WDR was 98 and 93% identical to its cowpea (*Vigna unguiculata*) and soybean (*G. max*) orthologs, respectively. The percent amino acid identity for the remainder of the species ranged from 84 (*Cicer arietinum* and *M. truncatulata*) to 62% (*Panicum hallii* and *Sorghum bicolor*). A phylogenetic tree (Fig. 3a) found that these identity values reflected their taxonomic distances from common bean (Supplementary Table 7). At the taxonomic order level, the NJ tree agrees with the current angiosperm tree (The Angiosperm Phylogeny Group 2016). Among the Fabales species, the gene tree is consistent with the most recent legume species tree based on multi-locus sequence data (Koenen *et al*. 2020).

Plant MYB proteins typically contain two conserved N terminal MYB domains, R2 and R3, and the R3 domain interacts with a bHLH protein. The variable C-terminal portion of the proteins is thought to provide the distinct functionality of each member of the family (Millard *et al*. 2019). These functions include anthocyanin and proanthocyanin biosynthesis, abiotic and biotic stress responses, cell fate, meristem and lateral organ development, hormone-induced growth, and embryogenesis (Dubos *et al*. 2010). To provide insight into the functionality of the *Z* MYB gene in color expression, the phylogeny presented here (Fig. 3b) focused on proteins experimentally shown to be associated with anthocyanin and proanthocyanin regulation (Supplementary Table 8). Based on a high bootstrap value, Z MYB was placed in a highly supported clade of genes previously shown to activate proanthocyanins production (Fig. 3b).

### T WDR and z MYB polymorphisms among common bean genotypes

A blastn analysis with the T WDR sequence as a query found orthologs in the three other common bean reference genomes: *Phvul.009G044700* (G19833; Schmutz *et al*. 2014), *PvUI111.09G045300* (UI 111), and *PvLabOv.09G054700* (Labor Ovalle) were identical at the protein level. The CDS sequence of the UI 111 and Labor Ovalle Middle American orthologs were identical to the 5-593 sequence and differed from the Andean ortholog G19833 sequence by two SNPs: T102C and C471A. Based on the fact that G19833 and 5-593 carry the dominant *T* allele (Bassett *et al*. 2011), it can be concluded that UI 111 and Labor Ovalle also carry the dominant *T* allele. This is consistent with the fact that neither of these genotypes has partially colored seeds.

The *T WDR* nucleotide sequence was determined for two recessive *tt* genotypes, Earliwax and 65–73 (Fig. 1, b and d). The recessive *t* allele of the 65–73 genotype contained an in-frame 39 nucleotide deletion (CDS:25-63) that results in a protein that lacks 13 amino acids near the N-terminal region (Supplementary Fig. 3). This allele was designated *t*^65–73^. A single nucleotide deletion at CDS position 752 of the genotype Earliwax resulted in a stop codon that reduced the protein length by 82 amino acids relative to the Pv5-593.09G047300 protein. This allele was designated *t*^EW^. A third recessive allele was detected in PI 632734. The allele is identical to *t*^EW^, except for an additional 5-bp deletion beginning at position 426 of the CDS. The mutation introduced a premature stop codon that shortened the protein to 178 amino acids. This allele was designated *t*^PI632734^.

A blastn analysis with the *Z MYB* against the other three other common bean reference genomes found the UI 111 sequence identical to the 5-593 sequence. The G19833 and Labor Ovalle orthologs contain 12 and 18 nucleotide (nt) in-frame deletions, respectively, that shorten their protein sequences by four and six amino acids, respectively (Supplementary Fig. 4). They both share a common SNP, while Labor Ovalle contains one non-synonymous and five additional synonymous SNPs. The G19833 allele was named *Z*^G19833^, and the Labor Ovalle allele was named *Z*^LO^.

The sequence of recessive *zz* donor lines V0869 (PI527806), V0919 (PI527820), and Earliwax (PI549618) used to develop multiple 5-593 introgression lines was extracted from a 40× resequencing data set (McClean *et al*. 2022). Relative to 5-593, all three lines share the 12 nt in-frame deletion with G19833. In addition, each line contained a non-synonymous G315T SNP of the CDS sequence, which led to a K105N amino acid substitution not found in G19833. This allele was designated as *z*^EW^. G19833 contains a colored hilum ring that is controlled by the dominant *Z* allele. Since it shares the 12 nt deletion with the three z donor parents, which lack the hilum ring, that deletion does not appear to be the functional mutation. Rather, the G315T SNP responsible for the K105N substitution in recessive *zz* lines appears to be the candidate causative mutation. This suggestion is further supported by the fact that the lysine at position 105 is conserved in all other functional MYB proteins associated with activators and repressors of proanthocyanin and anthocyanin synthesis described above (Fig. 3b).

### *T* and *Z* gene diversity among middle American and Andean bean genotypes

PACE markers specific to mutation *t*^65–73^ and *t*^EW^ alleles (Supplementary Table 4) were tested on a selection of genotypes Dr. Bassett used to create introgression lines homozygous recessive for *t* allele (Supplementary Table 1). Each line, whose *t* allele traced back to Earliwax, was positive for the *t*^EW^ PACE marker, while *t* introgression lines derived from 65–73 were positive for the *t*^65–73^ PACE marker allele. Members of the MDP (n = 284) and ADP (n = 256) populations were also screened with these markers. All MDP (Supplementary Table 2) and ADP (Supplementary Table 3) genotypes carried the dominant *T* allele at each of the two *T* markers. For the *Z* candidate gene, PACE markers for both the G315T SNP and the 12 nt deletion in the *z*^EW^ allele were developed. The dominant *Z* allele predominated in the MDP (93.3%), whereas a large majority of the ADP carried the recessive *z*^EW^ allele (96.8%).

### Genetic analysis of the *T* and *Z* genes

The linkage between the *T* PACE markers for *Pv5-593.09G047300* and the *Z* PACE markers and *Pv5-593.03G127600* (Supplementary Table 6) and their associated phenotypes were evaluated in F_2_ populations. The *t*^EW^ PACE marker co-segregated with flower color in the 4–68 F_2_ population (Brady *et al*. 1998). Every purple-flowered individual (n = 60) was either homozygous or heterozygous for the dominant 5-593 *T* marker allele, while all white flowered individuals (n = 19) were homozygous for the recessive *t*^EW^ marker allele. This provided additional genetic evidence that the *Pv5-593.09G047300* was a strong candidate gene for the *T* gene.

The linkage between the *Z* gene and the *Pv5-593.03G127600* z^EW^ marker was tested with three populations segregating for the Earliwax *z* allele. The 4–76 population segregated 3:1 for presence (*Z*_) or absence (*zz*) of the hilum ring. All F_2_ plants with a hilum ring (n = 63) were either homozygous or heterozygous for the dominant 5-593 *Z* marker allele, while those without a hilum ring (n = 11) were homozygous for the z^EW^ marker allele. The 6-160.161 F_2_ population segregated in the expected 3:1 ratio of self-colored (*ZZ*) + ambigua (*Zz*^EW^) seed patterns versus the virgarcus (*z*^EW^*z*^EW^) pattern. All individuals in the population expressing self-colored or ambigua pattern (n = 60) contained the 5-593 *Z* marker allele, while all virgarcus individuals (n = 19) were homozygous for the z^EW^ marker allele. For the third population, 6–273, the expected 3:1 ratio [self-colored (*ZZ*) + Anasazi (*Zz*^EW^) versus Anabip + virgarcus (*z*^EW^*z*^EW^)] was observed. All self-colored or Anasazi patterned plants (n = 53) carried the 5-593 *Z* marker allele, and those plants expressing the Anabip or virgarcus pattern (n = 19) were homozygous for the z^EW^ marker allele. The collective data across all three populations (n = 225) provide compelling evidence that *Pv5-593.03G127600* is a very strong candidate gene model for the *Z* gene. High-quality illustrations of each of these seed coat phenotypes can be found in Figs. 8.3 to 8.5 in Bassett (2007).

### Protein modeling of T, Z, and P

**Fig. 4.**
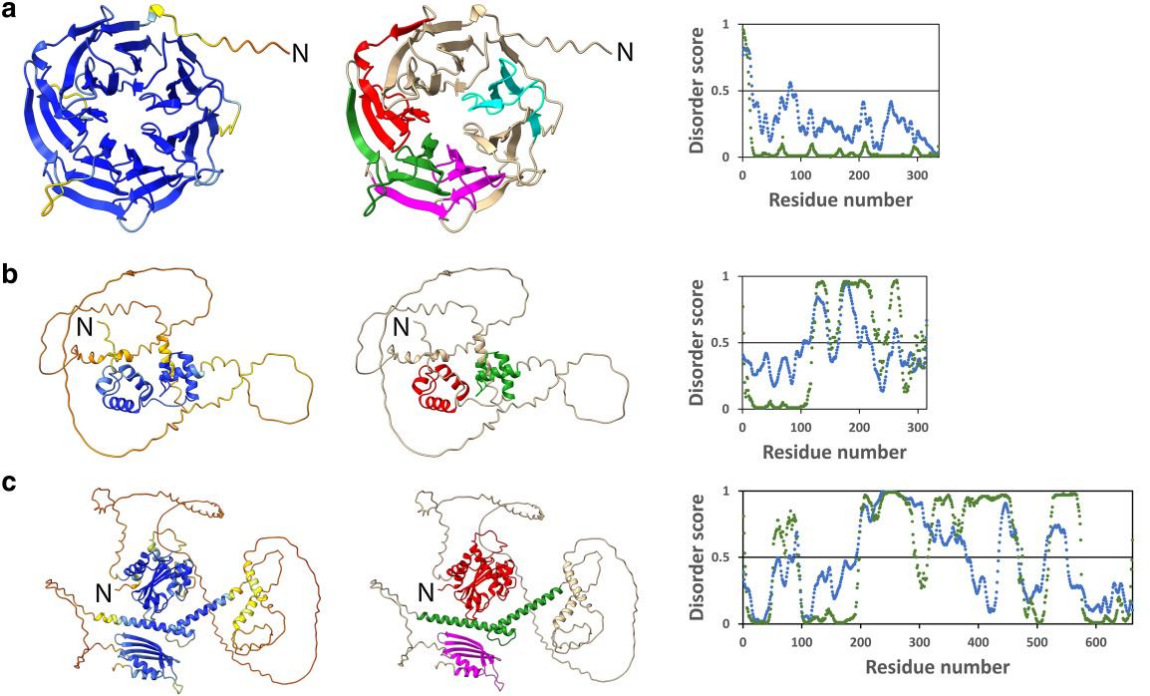
Structural details of the AlphaFold2 protein models of the a) *T* WDR, b) *Z* MYB, and c) *P* bHLH genes. For each protein, the left model is the pLDDT values across the protein. High pLDDT values for a residue suggest a good structural fit. Blue represents high confidence; yellow is moderate confidence; and red and orange are poor confidence. The central figures show the location of the domains in the proteins. For the *T* WDR protein a), the four WD40 domains are depicted. The R2 and R3 MYB domains are shown for the *Z* MYB protein b). The MYB-interacting region, helix–loop–helix, and activation domains are highlighted for *P* bHLH protein. Note that these domains were modeled with high confidence based on their pLDDT values. The right most image for each protein is the disorder score. The green represents the DISOPRED3 score, and the blue represents the IUPred3 score. Regions with disorder scores >0.5 are considered to be disordered. Note that the disorder regions also have low pLDDT values.

Domain analyses identified four WD-40 repeats (SMART accession number: SM00320; Pfam accession: PF00400) in the T WDR protein and its soybean (GmWD40), Medicago (MtWD40-1), and Arabidopsis (AtTTG1) orthologs (Supplementary Fig. 1). The bean T WDR AF2 model contained seven blades, each consisting of four anti-parallel β-strands labeled a, b, c, and d (Smith *et al*. 1999; Jain and Pandey 2018) to form the classic toroidal β-propeller structure of WDR proteins. As an indication of the AF2 model quality, the predictive score (pLDDT) for T WDR was >90 for more than 82% of the residues and >70 for more than 90% of the residues (Fig. 4a). This agrees with the DISOPRED3 and IUPre disorder analyses where only a few residues were considered disordered (Fig. 4a). The predicted structure of T WDR was in good agreement with the predicted structure of GmWD40, MtWD40-1, and AtTTG1 with C_α_ RMSD values of 0.714 Å, 1.431 Å, and 2.585 Å, respectively, across the full 336 residues.

Domain analysis of the Z MYB protein and its orthologs GmTT2B MYB, MyMYB14, and AtTT2 MYB revealed two MYB domains (SMART accession number: SM00717; Pfam accession: PF00249). Plant MYB proteins typically contain two tandem repeats each with three alpha-helices each arranged in helix–turn–helix motifs that are involved in DNA binding (Feller *et al*. 2011). Structure prediction by AF2 on the Z protein candidate contained the expected six N-terminal helices, three each for the R2 and R3 MYB domains (pLDDT > 90). The C-terminal region was primarily disordered and had low predicted scores (pLDDT < 60) (Fig. 4b). Transcription factors typically are disordered (Liu *et al*. 2006), and this was especially noted for plant MYB factors (Millard *et al*. 2019). DISOPRED3 and IUPred3 analyses of Z MYB found the protein to be primarily disordered, except for the MYB regions (Fig. 4b). The AF2 prediction found that the four amino acids essential for interaction with a target promoter in the Arabidopsis WER MYB protein [Pv Z residues: K51, N102, K105, N106; (Wang *et al*. 2020)] are appropriately positioned on the recognition helices of the R2 and R3 MYB domains. Further, the DxxxLxxRLxxLx_13_R motif shown to be responsible for the interaction between At WER MYB protein and its bHLH partner EGL3 is located in helix 1 of the R3 MYB domain (Wang *et al*. 2020). This, along with the sequence conservation and phylogenetic analysis, supports the role of Z MYB in the regulation of flavonoid production.

Modeling also included the P bHLH (Pv5-593.07G170300) protein. This model contains an MYB-interacting region (MIR), a helix–loop–helix (HLH), and ACT-like domains typical of bHLH proteins associated with regulation of flavonoid LBGs (McClean *et al*. 2018). Its AF2 model (Supplementary Fig. 4) identified four well-folded domains: the N-terminal MIR domain (residues 5-190) involved in bHLH/MYB protein interactions; a bHLH domain consisting of the basic residues 456–471; the HLH (residues 472–528) required for homo- or hetero dimerization with bHLH proteins (Feller *et al*. 2011); and the C-terminal ACT-like domain (residues 578–656), which is also involved in dimerization (Feller *et al*. 2006). The ACT-like domain has the characteristic ββαββα fold structure associated with plant bHLH proteins that regulate flavonoid biosynthetic genes (Kong *et al*. 2012). The conserved HER domain associated with binding of the protein to target promoters (Feller *et al*. 2011) is located in the basic region (463–471). All three domains are predicted with high confidence (pLDDT > 70) (Fig. 4c). The remaining protein was disordered with low confidence (pLDDT < 50). The DISOPRED3 and IUPred3 analyses agree with the AF2 prediction of disorder for residues between the folded domains (Fig. 4c). It was previously shown that the P protein functions as a master regulator of flavonoid expression in seed coats and was predicted to be part of a common bean MBW complex (McClean, *et al*. 2018).

The AF2 T WDR, Z MYB, and P bHLH AF2 structure predictions were compared with experimental crystal structures of similar proteins found in the protein databank (rcsb.org). The RMSD value between T WDR domain and the WDR domain of COP1 (At2g32950; PDB id: 6QTV), a seven-blade, four WD-40-domain protein that acts as a photomorphogenesis repressor (Lau and Deng 2012), was 1.219 Å (over 159 residues). The RMSD value between the Z MYB R2R3-MYB domains and the crystal structure of the R2R3-MYB domains of the WER (At5g14750; PDB id: 6KKS) that regulates epidermal cell fate (Lee and Schiefelbein 1999) was 0.827 Å (over 99 residues). Lastly, the RMSD value between the P MIR domain prediction and the N-terminal domain of Enhancer of Glabra 3 (EGL3) (At1g63659; PDB id: 7FDN) was 0.780 Å over 148 residues. EGL3 is the bHLH protein component of the Arabidopsis MBW complex regulating flavonoid biosynthesis (Gonzalez *et al*. 2008). Thus, the T WDR, Z MYB, and P bHLH AF2 models are in good agreement with experimental crystal structures of orthologs of the common bean proteins.

### Structural prediction for mutant T and Z alleles gene products

Computational protein modeling augments protein sequence data by defining structural differences that can explain the phenotypic effect of mutant alleles. t^65–73^ WDR lacks N-terminal residues 9–21 of the T WDR. Yet, AF2 predicts this protein still forms a seven β-propeller structure like T WDR. The key difference in the prediction for this mutant was the compensatory replacement of the d β-sheet of the seventh propeller with T WDR residues S3, T4, and Q5. Superposition of the t^65–73^ WDR model on the T WDR model gave an RMSD value of 0.674 Å (over 300 residues). Thus, it is likely there is little impact on protein stability as well as on any potential binding surfaces needed to function as the scaffold of the MBW complex.

The single base pair deletion of *t*^EW^ introduces an early stop codon after residue 254. The AF2 structure of t^EW^ WDR lacks the sixth and seventh blades from the β-propeller. Despite being able to form five blades, the t^EW^ WDR prediction did not close the propeller. Superposition of the *t*^EW^ model on the T WDR model gave an RMSD value 0.674 Å (over 254 residues). The loss of these two blades could have a significant impact on protein stability as well as potential binding surfaces needed to bind the βHLH or MYB components of the MBW complex.

A K105N amino acid substitution distinguished the mutant z^EW^ MYB AF2 structure from Z MYB (Supplementary Fig. 4). The lysine residue, invariant at this position across the phylogeny of functional MYB proteins associated with flavonoid biosynthesis (Fig. 3a), is critical in DNA binding, and mutating this residue in the WER protein “dramatically decreased” the interaction with its target promoter (Wang *et al*. 2020). The second difference is a four-residue deletion Q178-E181 found in the unstructured region of z^EW^ MYB.

### Computational modeling of a common bean MBW complex

To the best of our knowledge, an MBW complex has not been modeled previously to the extent available with AF2. Since previous research (McClean *et al*. 2018) and the results presented here strongly suggest that the *Z*, *P*, and *T* genes encode the MYB, bHLH, WDR proteins of a plant MBW complex associated with proanthocyanin biosynthesis, we decided to search for key interaction domains among the complex partners. Therefore, AF-Multimer was used to predict how these components could possibly assemble. To increase the potential reliability of these predictions, only residues with pLDDT values >70 for the three proteins were considered. We compared each protein structure modeled within the complex prediction to the corresponding monomer prediction. The structural predictions of Z MYB, P bHLH, and T WDR in the complex agreed well with the predictions of the monomer [RMSD = 1.077 (over 74 residues), 0.508 (over 104 residues), and 0.397 (over 336 residues), respectively]. We also predicted the soybean and Arabidopsis MBW protein complexes with AF-Multimer and compared each protein component in the modeled complex predictions to the corresponding individual protein predictions. For the soybean MBW complex, the GmTT2 MYB, GmTT8 bHLH, and GmTTG1 WDR proteins, the RMSD values were 0.996 (over 59 residues), 1.192 (over 84 residues), and 0.368 (over 301 residues, respectively. For the Arabidopsis MBW complex, the RMSD values for the AtTT2 MYB, AtTT8 bHLH, and AtTTG1 WDR proteins were 1.016 (over 7 residues), 0.474 (over 179 residues), and 0.483 (over 323 residues), respectively (n = 224).

**Fig. 5.**
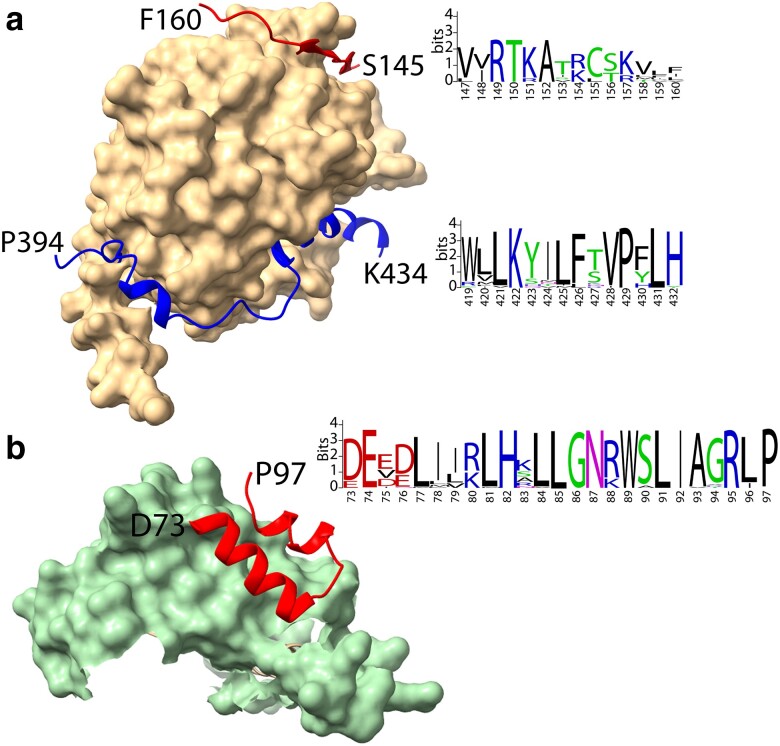
Interfaces between components of an AlphaFold-Multimer predicted model of a MBW complex of common bean. a) Interfaces between the T WDR protein (tan) and its interfacing regions with the Z MYB (red) and P bHLH (blue) proteins. To the right is a WebLogo representation of the sequences within the interface region that are highly conserved in other orthologs of the Z and P proteins. b) Interface between the MIR region of the P bHLH protein (green) and the Z MYB R3 domain (red). To the right is a WebLogo representation of the sequence within the interface region that is highly conserved among homologs of the Z protein. The vertical axis for sequences in A and B provides the WebLogo bit score (Crooks et al. 2004). The horizontal axis provides the amino acid positions of the domain in the full protein sequence.

The modeled Z MYB/P bHLH/T WDR MBW complex was analyzed with the PISA server to identify interacting regions. The Z MYB and T WDR interaction involves two β-strands formed by Z MYB residues 147–149 and 151–154, as well as strands βP3d (residues 161–167) and βP4d (residues 204–209) of T WDR (Fig. 5a). The two Z MYB β-strands fit the definition of a β-MoRF (beta-molecular recognition feature) since they are an intrinsically disordered region as a monomer and transitions to β structure in the presence of a binding partner (Mohan *et al*. 2006), in this case the T WDR protein. The same β-MoRF structural transition was also noted for the homologous residues in the predicted soybean GmTT2 MYB and Arabidopsis AtTT2 MYB proteins where they were modeled in their corresponding MBW complexes. The motif defined by the two β-strands of Z MYB is only found among MYB proteins experimentally confirmed to regulate PA synthesis (Fig. 3b) and has not been identified previously. This provides additional support of a potential function role of the motif in the binding MYB to WDR proteins in MBW complexes associated with PA biosynthesis. This Z MYB and T WDR interface accounts for 2.2% of the solvent accessible area (SAA) of Z MYB and 4.0% of the SAA of T WDR. The interaction is stabilized via hydrogen bonds involving Z MYB residues V148, T150, K151, and T153 and residues Q165, I167, E202, S204, and I206 of T WDR.

In the predicted MBW complex, P bHLH residues 395–431 wrap around T WDR starting at the propeller βP5–βP6 interface and across the top of the toroid axis and disengage at the top of the βP2–βP3 interface (Fig. 5a). The 419–432 region of P MYB contains highly conserved sequences among bHLHs that are components of other plant MBW complexes (Fig. 4c). These regions are well-supported helical structure with pLDDT values >70 (Fig. 4c). PISA analysis of this interaction indicates the P bHLH interface accounts for 11.7% of the SAA of the protein, while the SAA of the T WDR protein interface accounts for 10.1% of its total SAA. The contact residues of P bHLH with T WDR include two helices formed by residues 404–408 and residues 419–431. These two regions were also observed between GmTT8 bHLH and GmWD40 WDR in the predicted soybean MBW complex.

The interface between Z MYB and P bHLH involves Z MYB residues R3α1 and R3α2 within the R3 domain, which leaves R3α3 residues free to bind to DNA (Fig. 5b, Supplementary Fig. 2). P bHLH provides residues within helices α2, α3, and α5 within its predicted MIR domain for the interaction (Fig. 5b, Supplementary Fig. 5). Validation of the predicted model comes from its alignment with the interface found in the co-crystal structure of the At WER MYB and At EGL3 bHLH proteins (PDBID: 7FDL; Wang *et al*. 2022). The Z MYB and P bHLH interaction accounts for 4.0% of the Z MYB SAA and 2.1% of the P bHLH SAA. The interface regions contain two salt bridges: R88 of Z MYB and E98 of P bHLH and R95 of Z MYB and E 102 of P bHLH. The Z interface involves the highly conserved R3 domain, which has a similar structure in all experimentally determined flavonoid-activating MYB proteins that interact with a homolog of P bHLH. Ten Z MYB R3 residues are invariant in the MYB homologs. Of the Z MYB residues predicted to interact with P bHLH, five (E74, L85, L91, R95, P97) are invariant in Z MYB orthologs (Fig. 5b).

### Modeling of the P bHLH homodimer and Z MYB/P bHLH heterodimer

Since transcription factors, including bHLHs, are known to function as dimers (Murre *et al*. 1989), AF-Multimer was used to predict the homodimeric structure of the P protein. AF-Multimer enforces a global C2 symmetry axis. Therefore, all dimeric interfaces were placed along a single C2 axis. However, the large disordered regions between each of the dimer interfaces do not structurally predicate that there is a single global axis, but more likely, three individual local 2-fold symmetry axes. Thus, the individual interfaces are likely valid, but their location in three-dimensional space relative to each other is not. For example, the classic bHLH dimer is defined by a helical region that binds DNA, followed by a leucine zipper that forms the dimer interface. P bHLH residues 459–484 bind DNA and residues 493–528 contain the leucine zipper (Supplementary Fig. 5). However, a second dimer interaction between symmetrically related helical residues 416–432 is modeled in the DNA binding space, which would preclude the DNA binding function. Interactions between ACT-like domains mediate bHLH homodimer formation (Feller *et al*. 2006). Indeed, a third dimer interface was predicted to be formed between the two ACT-like domains. This interface is similar to that found for other ACT domains, such as in *Escherichia coli* phosphoglycerate dehydrogenases [PDB IDs: 1PSD, (Schuller *et al*. 1995); 1YBA (Thompson *et al*. 2005)]. Further support of this interface is that P bHLH residue V615, which is buried at this interface, corresponds positionally to a critical residue that determines the strength of the ACT-like domain interaction (Lee *et al*. 2021). However, the position of the ACT-like domains relative to the rest of the complex is defined by this interface residing on the global C2 axis. Therefore, while each interface may exist, the local relationships of the individual C2 axes is likely asymmetric, and reliable conclusions are difficult to discern relative to the orientations of the domains.

### Effects of mutant Z MYB and T WDR proteins on common bean MBW complex interactions

Protein interactions involving MBW complexes containing mutant z^EW^ MYB or t^EW^ WDR were evaluated using PISA. The model predicted that MYB R3 helix 3 residues 107–115 of z^EW^ MYB interact with T WDR WD40 domain 2 residues 119–127 as part of the mutant, but not the wild-type MBW complex. While both of these regions are well predicted (80 > pLDDT > 60), closer examination of this interface reveals the presence of several severe steric clashes that preclude this interface from being real. Therefore, it is unsurprising that this interaction is not observed in the wild-type Z MYB/P bHLH/T WDR complex. However, the z^EW^ K105N substitution would severely disrupt z^EW^ from interacting with its target DNA promoter. In the crystal structure of WT At WER MYB complexed with DNA (PDB ID:6KKS), K105 forms extensive interactions in the major groove of the DNA (Wang *et al*. 2020). The equivalent residue in z^EW^, N105, would lack these major groove interactions. Wang *et al*. (2020) found that this MYB region was important to promoter binding. The interaction between P bHLH and T WDR in the presence of z^EW^ remains largely unchanged from that predicted for the wild-type complex where P bHLH residues 395–431 wrapped around T WDR. However, there is an additional helical region defined by P bHLH residues 361–379 that is modeled with high confidence (92 > pLDDT >72). This region interacts with propeller strands βP4 and βP5 of T WDR and completes an offset belt that wraps around the T toroid.

A major structural effect of the C-terminal deletion of t^EW^ WDR was the elimination of the sixth and seventh blades from the β-propeller, thus forming an unclosed five bladed structure. While five-bladed propellers are possible, the inability of t^EW^ WDR to close leaves exposed hydrophobic surfaces that provide a strong driving force for false protein interfaces. Indeed, the ACT-like domain from P bHLH tries to extend the β-structure at the edge of propeller βP5. While this interface is modeled with high confidence, it likely relies on the extended beta structure. Before any reliable conclusions can be drawn from this predicted model, experiments would need to be performed to validate these non-physiological interfaces.

## Conclusion

In common bean, multiple genes (*G*, *B*, *V*, *R*, *Rk*, *Sal*) control seed coat color, while *T* and *Z* are two genes that control the spatial pattern of seed coat color expression. This led to the working hypothesis that these two genes were regulators of the flavonoid biosynthetic pathway. Physical intervals containing *T* and *Z* were determined based on previous marker data and the relationship between the genetic and physical distances of the regions, in which the markers were located in the common bean genome. Multiple analyses supported the conclusion that the *T* encodes a WDR protein, and *Z* encodes an MYB protein, and that these genes are critical to flavonoid synthesis in common bean. Domain and protein modeling found that the *Z* gene candidate encodes a mostly unstructured R2R3 MYB protein, while the *T* gene candidate encodes a highly structured, seven-blade WDR protein with four WD40 domains. Phylogenetic analysis found that these two proteins clustered with other WDR and MYB proteins experimentally shown to regulate gene expression of the flavonoid pathway. In addition, the previously cloned P bHLH protein was found to be highly similar to other bHLH proteins that function as a component of other defined plant MBW complexes (Payne *et al*. 2000; Zhang *et al*. 2003).

Structural models of individual MBW complex proteins are rare, and no structure model exists for an entire MBW complex. Only a single experimentally derived crystal model consisting of fragments of the WER R2R3 MYB (Wang *et al*. 2020) and EGL3 bHLH proteins (Wang *et al*. 2022) has been reported. We used AlphaFold2 to model the bean MBW proteins and to develop the first computationally derived structural model of a plant MBW complex. RMSD analyses of AF2 models of Z MYB and P bHLH found that these proteins are good structural fits with WER and EGL3, respectively, two components of an Arabidopsis MBW complex. The modeling of the bean MBW revealed multiple new discoveries. The complex interface of Z MYB and T WDR involved a potential β-MoRF in a Z MYB motif that is unstructured in the monomer state. This motif is conserved among plant proteins that regulate PA biosynthesis. This motif has not been described previously. Another first is the discovery of a P bHLH and T WDR interface that involves a P bHLH structured region that is conserved among other bHLH proteins involved in the regulation of PA via the MBW complex. Conversed MBW protein interactions were observed. Z MYB and P bHLH interact in the same regions as predicted from crystal structure analysis of Arabidopsis WER and EGL3 (Wang *et al*. 2022). The observation that Z MYB and P bHLH interact with different regions of T WDR in bean, soybean, and Arabidopsis complexes suggests that complex structure is not entirely conversed, and phenotypic function is plastic relative to complex structure. We also present the first computational models of MBW complexes containing a mutated partner. In the complex containing the t^EW^ WDR protein, the t^EW^ interaction with P bHLH was compromised by the deletion of the βP6 blade, which resulted in P binding elsewhere to T WDR. The introduction of the z^EW^ MYB protein in the complex created a new interaction that would comprise the ability of z^EW^ MYB to function. All of the new structural predictions can act as hypotheses for further yeast two hybrid and crystal structural analyses.

## Supplementary Material

jkae184_Supplementary_Data

## Data Availability

The NCBI T protein identifiers (genotype in parentheses) are OR500718(PI 451802), OR500719 (PI 549618), OR500720 (PI 632734), and OR500721 (PI 608674). The NCBI Z proteins identifiers (genotype in parentheses) are OR475097 (PI 549618) and OR475098 (PI 608674). Data supporting this research can be found within the main body of the paper or the Supplementary data published online. Supplemental material available at G3 online.
