# Supplementary material for: T and Z, partial seed coat patterning genes in common bean, provide insight into the structure and protein interactions of a plant MBW complex
Source: G3 (Bethesda). 2024 Aug 21;14(10):jkae184. doi: 10.1093/g3journal/jkae184 (PMC11457125; doi:10.1093/g3journal/jkae184)
Supplement: jkae184_Supplementary_Data [file jkae184_supplementary_data.zip › Figure_S1_G3-2024-405237.pdf]

```
                              βP7D      βP1A           βP1B
                          HHH βββββ    ββββββ          ββββββ
Pv-T         1 MDNSTQDSHLRSEN-SVTHESPYPIYAMAFSP----SHPHRLALGSFIEK 45
                          HHH βββββ    βββββ           ββββββ
GmWD40       1 MENSTEESHLRSEN-SVTYESPYPIYGMSFSP----SHPHRLALGSFIEE 45
                              βββββ    βββββ           ββββββ
MtWD40-1     1 MDNSTQESHLRSDNNSVTYDSPYPLYAMSISPNTNS-PQQRIAVGSFIEE 49
                              βββββ    βββββ          βββββββ
Pea-A2       1 MDNSTQESHLRSDNNSVTYDSPHPLYAMAFSSNPNPQHHQRIAVGSFIEE 50
                          HHH βββββ    ββββββ         βββββββ
AtTTG1       1 MDNSAPDSLSRSET-AVTYDSPYPLYAMAFSS-LRSSSGHRIAVGSFLED 48

                                             WDR1
                   βP1C       βP1D             βP2A
                ββββββββββ    ββββ HHH βββββ   ββββ             β
Pv-T        46 YTNRIHILSFHPDTLSITPHPSLSFDHPYPPTKLMFHPRKPS--PSSSSD 93
                Bβββββββββ    ββββ HHH βββββ   βββ              β
GmWD40      46 YNNRVDILSFHPDTLSVTPHPSLSFDHPYPPTKLMFHPRKPS--PSSSSD 93
                ββββββββββ    ββββ HHH ββββ    ββββ             β
MtWD40-1    50 YTNRIDILNFNPDTLSLKPQPSLSFDHPYPPTKLMFHPATHSSLQKTSSD 99
                ββββββββββ    ββββ HHH ββββ    ββββ      HHHH   β
Pea-A2      51 YTNRVDILSFNPDTLSIKPQPSLSFDHPYPPTKLMFHPATHSSLQKTSSD 100
                ββββββββββ    ββββ HHH ββββ    ββββ             β
AtTTG1      49 YNNRIDILSFDSDSMTVKPLPNLSFEHPYPPTKLMFSP--PSLRRPSSGD 96

                                               WDR2
               βP2B     βP2C        βP2D              βP3A
               βββββ   ββββββ      βββββββ           ββββββ
Pv-T        94 LLATSGDYLRLWDVREN--SVEPLSLFNNSKTSEFCAPLTSFDWNDIDHN 141
               βββββ   ββββββ      βββββββ           ββββββ
GmWD40      94 LLATSGDYLRLWEIRDN--SVDAVSLFNNSKTSEFCAPLTSFDWNDIDPN 141
               βββββ   ββββββ      βββββββ           ββββββ
MtWD40-1   100 LLATSGDYLRLWEVREN--SVEALSLFNNSKTSEFCAPLTSFDWNEIEPK 147
               βββββ   ββββββ      βββββββ           ββββββ
Pea-A2     101 LLATSGDYLRLWEVREN--SVEALSLFNNSKTSEFCAPLTSFDWNEIEPK 148
               βββββ   βββββββ     ββββββββ          ββββββ
AtTTG1      97 LLASSGDFLRLWEINEDSSTVEPISVLNNSKTSEFCAPLTSFDWNDVEPK 146

                                                  WDR3
                βP3B     βP3C      βP3D        βP4A      βP4B
               ββββββ   ββββββ    ββββββ      ββββββ    ββββββ
Pv-T       142 RIGTSSIDTTCTIWDIERTVVETQLIAHDKEVYDIAWGEARVFASVSADG 191
               ββββββ   ββββββ    ββββββ      ββββββ    ββββββ
GmWD40     142 RIATSSIDTTCTIWDIERTLVETQLIAHDKEVYDIAWGEARVFASVSADG 191
               ββββββ   ββββββ    βββββββ     ββββββ    ββββββ
MtWD40-1   148 RIGTSSIDTTCTIWDIERGVVETQLIAHDKEVYDIAWGESRVFASVSADG 197
               ββββββ   ββββββ    βββββββ     ββββββ    ββββββ
Pea-A2     149 RIGTSSIDTTCTIWDIERGVVETQLIAHDKEVYDIAWGESRVFASVSADG 198
               ββββββ   ββββββ    βββββββ     ββββββ    ββββββ
AtTTG1     147 RLGTCSIDTTCTIWDIEKSVVETQLIAHDKEVHDIAWGEARVFASVSADG 196

                βP4C        βP4D         βP5A        βP5B
               ββββββ      ββββββ       ββββββ      ββββββ     ββ
Pv-T       192 SVRIFDLRDKEHSTIIYESPHPDTPLLRLAWNKQDLRYMATILMDSNKVV 241
               ββββββ      ββββββ       ββββββ      ββββββ     ββ
GmWD40     192 SVRIFDLRDKEHSTIIYESPHPDTPLLRLAWNKQDLRYMATILMDSNKVV 241
               ββββββ      ββββββ       ββββββ      ββββββ     ββ
MtWD40-1   198 SVRIFDLRDKEHSTIIYESPQPDTPLLRLAWNKKDLRYMATILMDSNKVV 247
               ββββββ      ββββββ       ββββββ      ββββββ     ββ
Pea-A2     199 SVRIFDLRDKEHSTIIYESPQPDTPLLRLAWNKKDLRYMATILMDSNKVV 248
               ββββββ      ββββββ       ββββββ      ββββββ     ββ
AtTTG1     197 SVRIFDLRDKEHSTIIYESPQPDTPLLRLAWNKQDLRYMATILMDSNKVV 246

                                            WDR4
               βP5C       βP5D      βP6A        βP6B      βP6C
               βββ        βββ      ββββββ      ββββββ   ββββββ
Pv-T       242 ILDIRSPTTPVAELERHRGSVNAIAWAPHSSTHICSAGDDTQALIWELPT 291
               βββ        βββ      ββββββ      ββββββ   ββββββ
GmWD40     242 ILDIRSPTTPVAELERHRGSVNAIAWAPHSSTHICSAGDDTQALIWELPT 291
               βββ        βββ      ββββββ      ββββββ   ββββββ
MtWD40-1   248 ILDIRSPTTPVAELERHRAGVNAIAWAPRSSKHICSGGDDAQALIWELPA 297
               βββ        βββ      ββββββ      ββββββ   ββββββ
Pea-A2     249 ILDIRSPTTPVAELERHRAGVNAIAWAPRSSKHICSAGDDTQALMWELPT 298
               βββ        βββ      ββββββ      ββββββ   ββββββ
AtTTG1     247 ILDIRSPTMPVAELERHQASVNAIAWAPQSCKHICSGGDDTQALIWELPT 296

                         βP6D      βP7A          βP7B    βP7C
                         ββββ      ββββ      ββββββ  ββββββ
Pv-T       292 LAAPSGIDPMCMYSAGCEINQLQWSAAQPDWIAIAFANKVQLLKV 336
                         ββββ      ββββ      ββββββ  ββββββ
GmWD40     292 LASPTGIDPVCMYSAGCEINQLQWSAAQPDWIAIAFANKMQLLKV 336
                         ββββ    ββββββ      ββββββ  ββββββ
MtWD40-1   298 VAGPNGIDPMTTYSAGCEINQLQWSAAQPDWIAIAFANKMQLLRV 342
                         ββββ      ββββ      ββββββ  ββββββ
Pea-A2     299 VAGPNGIDPMSMYSSCYEINQLQWSAAQPDWIAIAFANKMQLLRV 343
                         ββββ    ββββββ      ββββββ  ββββββ
AtTTG1     297 VAGPNGIDPMSVYSACSEINQLQWSSSQPDWIGIAFANKMQLLRV 341
```
