# Supplementary material for: T and Z, partial seed coat patterning genes in common bean, provide insight into the structure and protein interactions of a plant MBW complex
Source: G3 (Bethesda). 2024 Aug 21;14(10):jkae184. doi: 10.1093/g3journal/jkae184 (PMC11457125; doi:10.1093/g3journal/jkae184)
Supplement: jkae184_Supplementary_Data [file jkae184_supplementary_data.zip › Supplemental_Material_Legends_G3-2024-405237.docx]

**Table S1**. Select Bassett introgression lines genotyped with the tEW and t65-73 PACE markers.

**Table S2**. T and Z genotypes of members of the Middle American Diversity Panel.

**Table S3**. T and Z genotypes of members of the Andean Diversity Panel.

**Table S4**. Sequences of primers used to amplify amplicons from the T gene.

**Table S5**. The sequences of the PACE primers used to genotype the tEW and t65-73 alleles of the T gene.

**Table S6**. T and Z populations used to map candidate genes.

**Table S7**. Sequence IDs of a representative sample of higher plant T protein orthologs used for the phylogenetic analysis. The E-value and % identity values are from a blastp analysis using Pv5-593.09G047300 query against each of the Phytozome reference genomes to identify the corresponding ortholog,

**Table S8**. Protein sequences used for the phylogenetic analysis of Z gene candidate (Pv5-593.03G127600) and proteins known to act as activators or repressors of proanthocyanin or anthocyanin biosynthesis. The E-value and % identity values are from a blastp analysis using Pv5-593.03G127600 as a query against the MYB proteins to calculate E-value and percent identity.

**Figure S1.** Multiple sequence alignment of WDR proteins of common bean *T* (Pv5-593.09G047300), soybean GmWD40, *M. truncatula* MtWD40-1, pea A2, and *A. thaliana* TTG1. The locations of α-helices and β-sheets, as predicted by AF2, are designated as consecutive “H”s and “β”s. Each β-propeller blade is designated βP#, and each β-sheet within a blade is designated a, b, c, or d. The position of each WD-40 repeat (WDR) is shown.

**Figure S2**. Multiple sequence alignment of common bean *Z* (Pv5-593.03G127600), soybean GmTT2B, *M. truncatula* MtMYB14, and *A. thaliana* AtTT2. The locations of α-helices, as predicted by AF2, are designated as consecutive “H”s. R2 and R3 designated the two MYB domains, and H1, H2, and H3 designated the helices in each of the MYB domains. Conserved Arabidopsis MYB domain residues experimentally shown to interact with DNA of a target promoter (Wang *et al.* 2020) are shown with an X.

**Figure S3.** Structure of wild type (Pv-T) and mutant (Pv-t^65-73^ and Pv-t^EW^) proteins. The mutant proteins are associated with partial seed color and white flowers. The locations of α-helices and β-sheets, as predicted by AF2, are designated as consecutive “H”s and “β”s. Each β-propeller blade is designated βP#, and each β-sheet with a blade is designated a, b, c, or d.

**Figure S4.**  Structure of common bean wild type (Pv-Z, Pv-Z^G19833^, and Pv-Z^LO^) and mutant (Pv-z^EW^) proteins. The locations of α-helices, as predicted by AF2, are designated as consecutive “H”s. R2 and R3 designate the two MYB domains, and H1, H2, and H3 designate the helices in each of the MYB domains. Conserved MYB domain residues experimentally shown to interact with DNA of a target promoter (Wang *et al.* 2020) are shown with an X. Conserved residues that interact with a bHLH partner are shown with an * (Wang *et al.* 2022).

**Figure S5.** Common bean wild type P protein sequence, domain boundaries and AlphaFold2 predicted secondary structure elements. The MYB-MIR-interacting region (MIR) is highlighted in cyan, the bHLH basic region highlighted in green, the helix-loop-helix (HLH) domain is highlighted in yellow, and the activation domain (ACT) is highlighted in grey. The three asterisks below the basic region are the Hx_3_Ex_3_R motif associated with binding to target promoters. Secondary structure elements are indicated above the sequence with residues predicted to form helical structure designated by Hs, and residues predicted to form beta strands notated by consecutive β symbols.
