# Supplementary figures and images for: T and Z, partial seed coat patterning genes in common bean, provide insight into the structure and protein interactions of a plant MBW complex
Source: G3 (Bethesda). 2024 Aug 21;14(10):jkae184. doi: 10.1093/g3journal/jkae184 (PMC11457125; doi:10.1093/g3journal/jkae184)

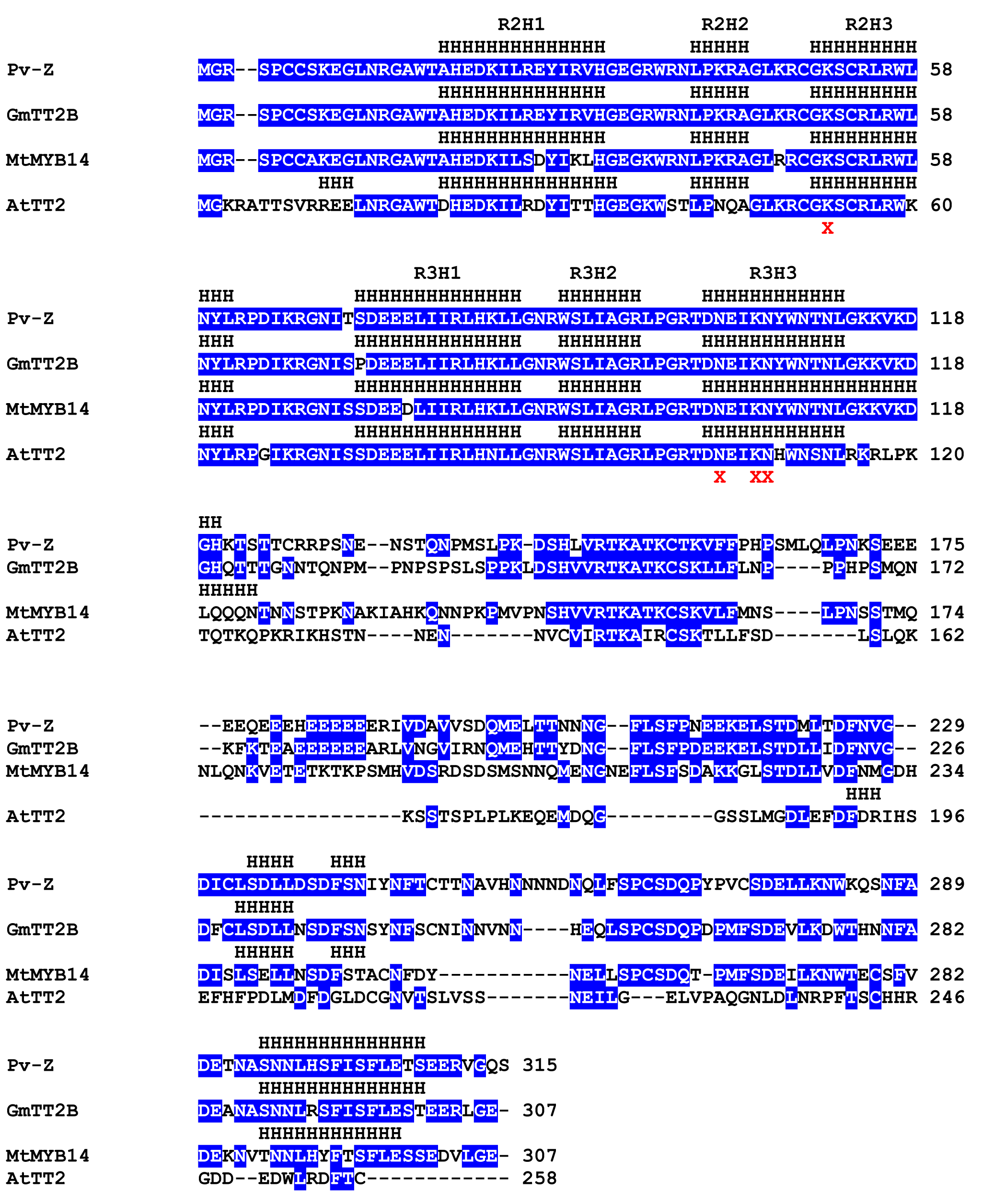

Supplement: jkae184_Supplementary_Data [file jkae184_supplementary_data.zip › Figure_S2_G3-2024-405237.tif]

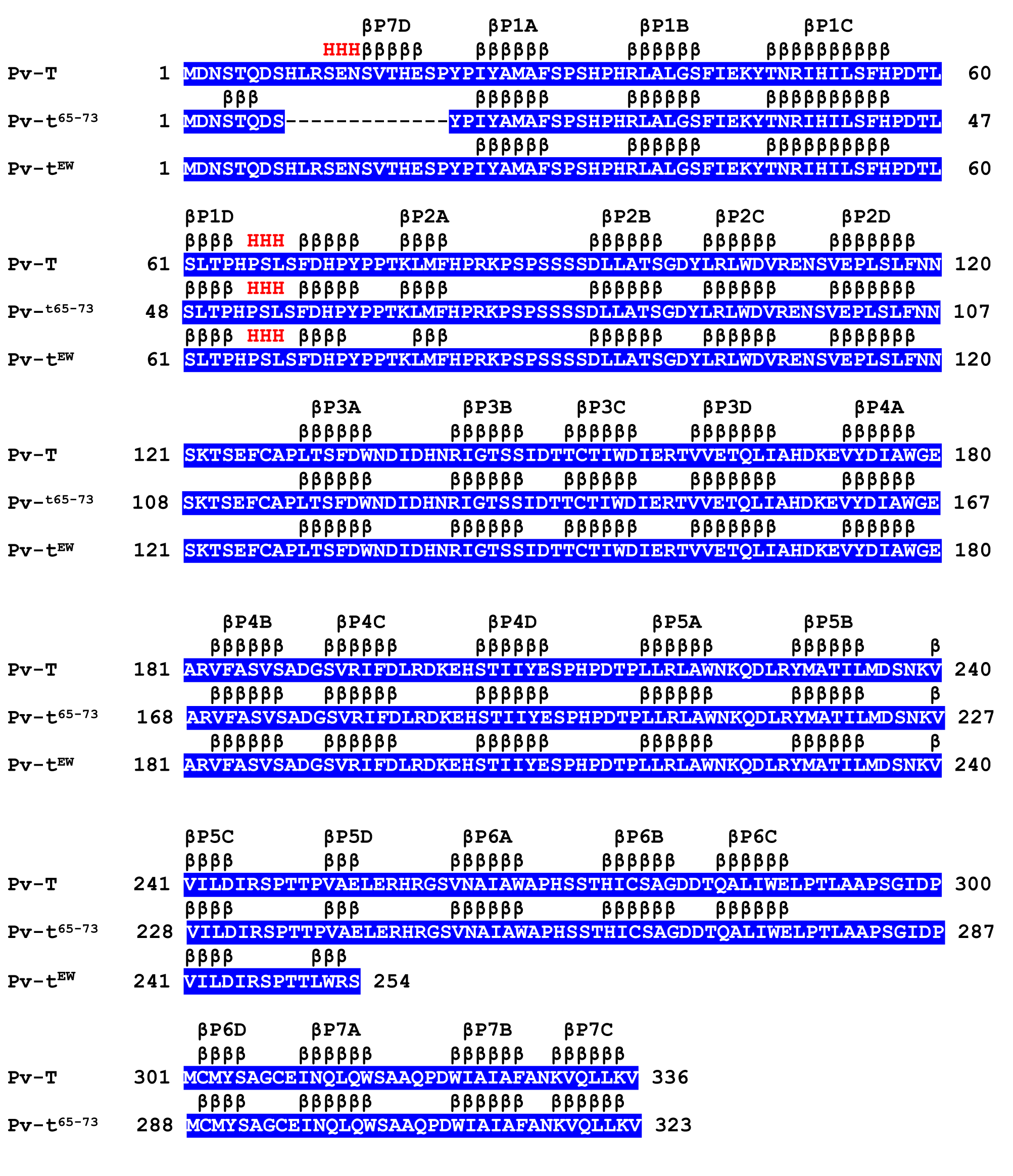

Supplement: jkae184_Supplementary_Data [file jkae184_supplementary_data.zip › Figure_S3_G3-2024-405237.tif]

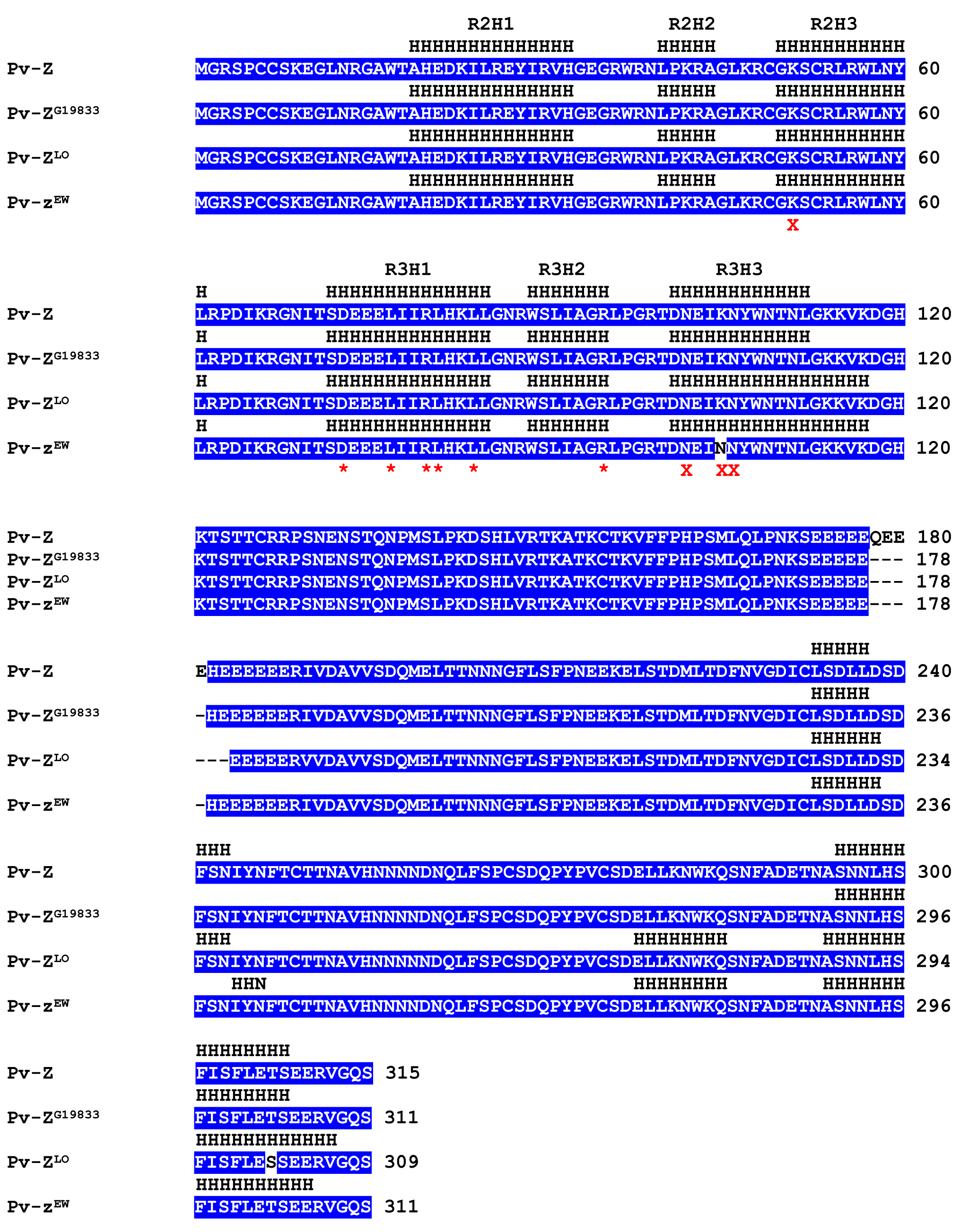

Supplement: jkae184_Supplementary_Data [file jkae184_supplementary_data.zip › Figure_S4_G3-2024-405237.tif]

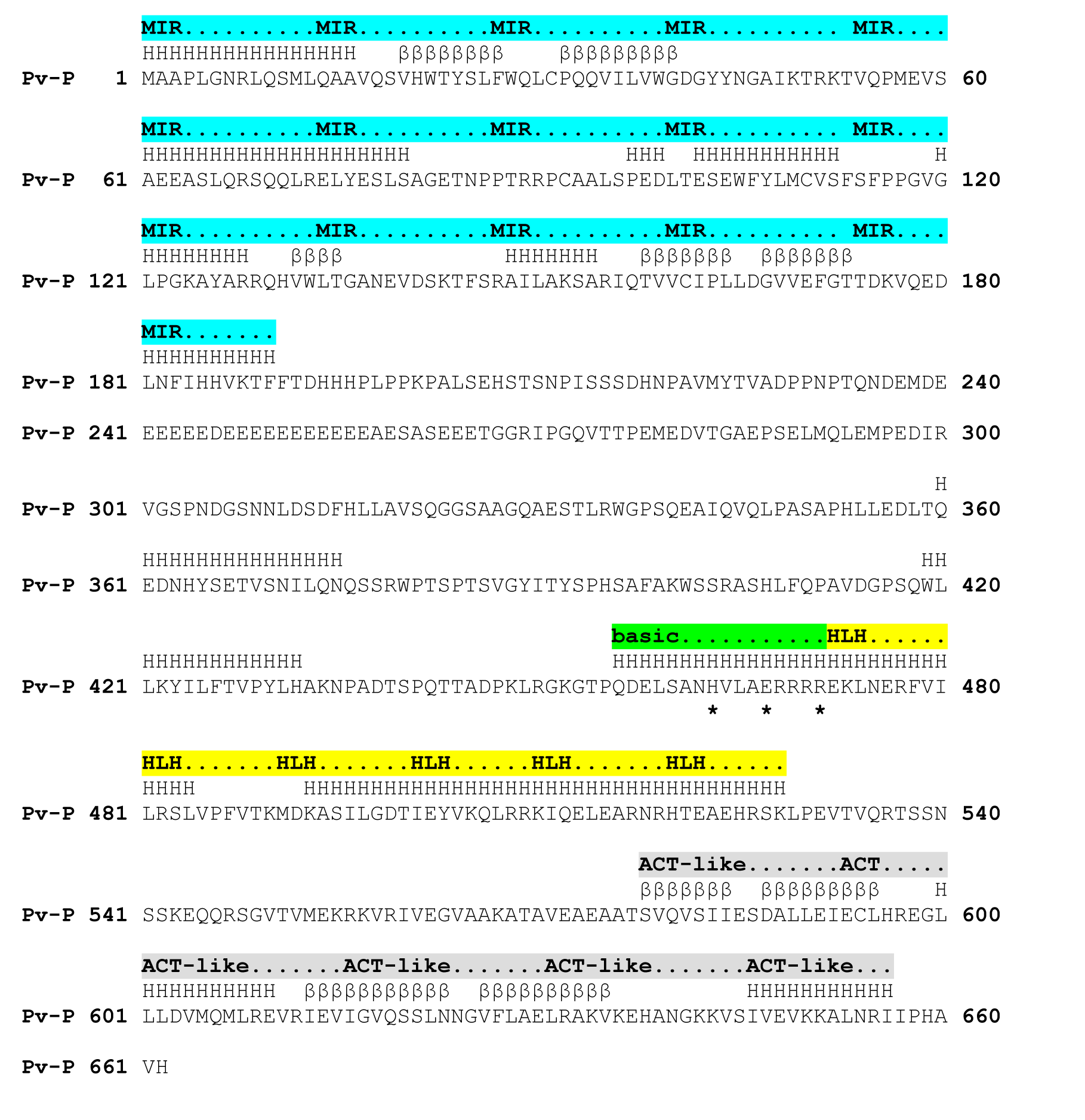

Supplement: jkae184_Supplementary_Data [file jkae184_supplementary_data.zip › Figure_S5_G3-2024-405237.tif]
